# Modified delta-shaped gastroduodenostomy consisting of linear stapling and single-layer suturing with the operator positioned between the patient’s legs: A technique preventing intraoperative duodenal injury and postoperative anastomotic stenosis

**DOI:** 10.1371/journal.pone.0230113

**Published:** 2020-03-06

**Authors:** Takaya Tokuhara, Eiji Nakata, Toshiyuki Tenjo, Isao Kawai, Keisaku Kondo, Shigeru Hatabe

**Affiliations:** Department of Surgery, Otori Stomach and Intestines Hospital, Osaka, Japan; Melbourne University, AUSTRALIA

## Abstract

**Background:**

The drawback of the delta-shaped gastroduodenostomy (DSG) in totally laparoscopic distal gastrectomy (TLDG) is the presence of intraoperative duodenal injury and postoperative anastomotic stenosis, which can occur due to a relatively short duodenal bulb diameter.

**Materials and methods:**

From June 2013 to June 2019, 35 patients with gastric cancer underwent TLDG with a modified DSG consisting of linear stapling and single-layer hand suturing in our institution. All anastomotic procedures were performed by the right hand of the operator positioned between the patient’s legs. Linear stapling of the posterior walls of the remnant stomach and duodenum without creating a gap was performed using a 45-mm linear stapler, considering the prevention of intraoperative duodenal injury. The stapler entry hole was closed using a single-layer full-thickness hand suturing technique with knotted sutures and a knotless barbed suture. We described the clinical data and outcomes in the present retrospective patient series.

**Results:**

No intraoperative duodenal injury occurred in any of the 35 patients. The median staple length at linear stapling of the posterior walls of the remnant stomach and duodenum was 41.7 ± 4.2 (30–45) mm, and 2 patients (5.7%) had a staple length of 30 mm. There were no incidences of postoperative anastomotic stenosis.

**Conclusions:**

We suggest that a modified DSG consisting of linear stapling and single-layer hand suturing performed by an operator positioned between the patient’s legs can be one option for B-Ⅰ reconstruction following TLDG because it can aid in preventing both intraoperative duodenal injury and postoperative anastomotic stenosis.

## Introduction

Over the past 20 years, totally laparoscopic distal gastrectomy (TLDG), in which all procedures including lymph node (LN) dissection, resection of the stomach and duodenum, and anastomosis are performed laparoscopically, has been developed thanks to improvements in the instruments and techniques. Among the intracorporeal anastomotic techniques, Billroth Ⅰ (B-Ⅰ) reconstruction is performed when both the size of the remnant stomach and the length of the duodenal bulb are sufficient, and the delta-shaped gastroduodenostomy (DSG) as a B-Ⅰ reconstruction method in TLDG, in which a functional end-to-end anastomosis is performed using only 45-mm endoscopic linear staplers, has been accepted worldwide [1–13]. In this technique, after the duodenum is transected in a posteroanterior direction and a sufficient space for stapler insertion is created around the posterior wall of the duodenal bulb, stapling of the posterior walls of the remnant stomach and duodenum using one linear stapler is performed. Thereafter, the stapler entry hole is closed using two linear staplers. All of these staplings are performed by the left hand of the assistant positioned at the left side of the patient under the guidance of the operator positioned at the right side of the patient.

However, although the incidence is low, one drawback of the DSG is the occurrence of intraoperative duodenal injury due to stapler insertion when performing linear stapling of the posterior walls of the remnant stomach and duodenum [11]. When this complication occurs, additional resection of the duodenum and conversion to other techniques including Roux-en-Y (R-Y) or Billroth Ⅱ reconstruction needs to be performed. Intraoperative duodenal injury in the DSG is thought to occur when the diameter of the duodenal bulb is relatively short, and one reason for this complication is that the stapling is performed by the assistant’s left hand. In addition, another drawback of the DSG is the occurrence of postoperative anastomotic stenosis, although again, the incidence is low [2, 12]. Similar to intraoperative duodenal injury, postoperative anastomotic stenosis following the DSG can occur when the diameter of the duodenal bulb is relatively short because the staple length at linear stapling of the posterior walls of the remnant stomach and duodenum is not sufficient and the stapler entry hole is closed using the linear stapler.

From June 2013 to June 2019, 35 patients with gastric cancer underwent a modified DSG consisting of linear stapling and single-layer hand suturing with the operator positioned between the patient’s legs following TLDG in our institution. We describe the clinical data and outcomes in this patient series retrospectively, including the incidence of intraoperative duodenal injury and postoperative anastomotic stenosis.

## Materials and methods

### Patients

In June 2013, TLDG with B-Ⅰ or R-Y reconstruction was introduced in our institution. The indication for TLDG at our institution is clinical stage Ⅰ gastric cancer, according to the Japanese classification of gastric carcinoma [14], that is located in the middle or lower third of the stomach and is not a candidate for endoscopic submucosal dissection [15]. B-Ⅰ reconstruction was used when both the size of the remnant stomach and the length of the duodenal bulb were sufficient, and gastroesophageal reflux due to hiatal hernia was not anticipated. Antecolic R-Y reconstruction with an antiperistaltic gastrojejunostomy was used when the remnant stomach was small, the length of the duodenal bulb was short, or gastroesophageal reflux due to hiatal hernia was anticipated [4, 16–18].

From June 2013 to June 2019, 35 patients (20 men and 15 women) with gastric cancer underwent a modified DSG consisting of linear stapling and single-layer hand suturing with the operator positioned between the patient’s legs following TLDG in our institution. Written informed consent was signed by each patient who agreed to undergo TLDG. All of these patients were checked regularly in the outpatient care after discharge, which included physical examination, laboratory tests, examination with a 9.6-mm-diameter endoscope (EG-590WR2; FUJIFILM Co., Ltd., Tokyo, Japan), chest CT, and abdominal CT. Postoperative complications were recorded according to the Clavien-Dindo classification [19]. We described the clinical data and outcomes in the present patient series retrospectively. The individual described in this article has given written consent to publish these case details. This retrospective study was approved by the Institutional Review Board of the Otori Stomach and Intestines Hospital (approval no. 19000009).

### Surgical procedure

**Fig 1:**
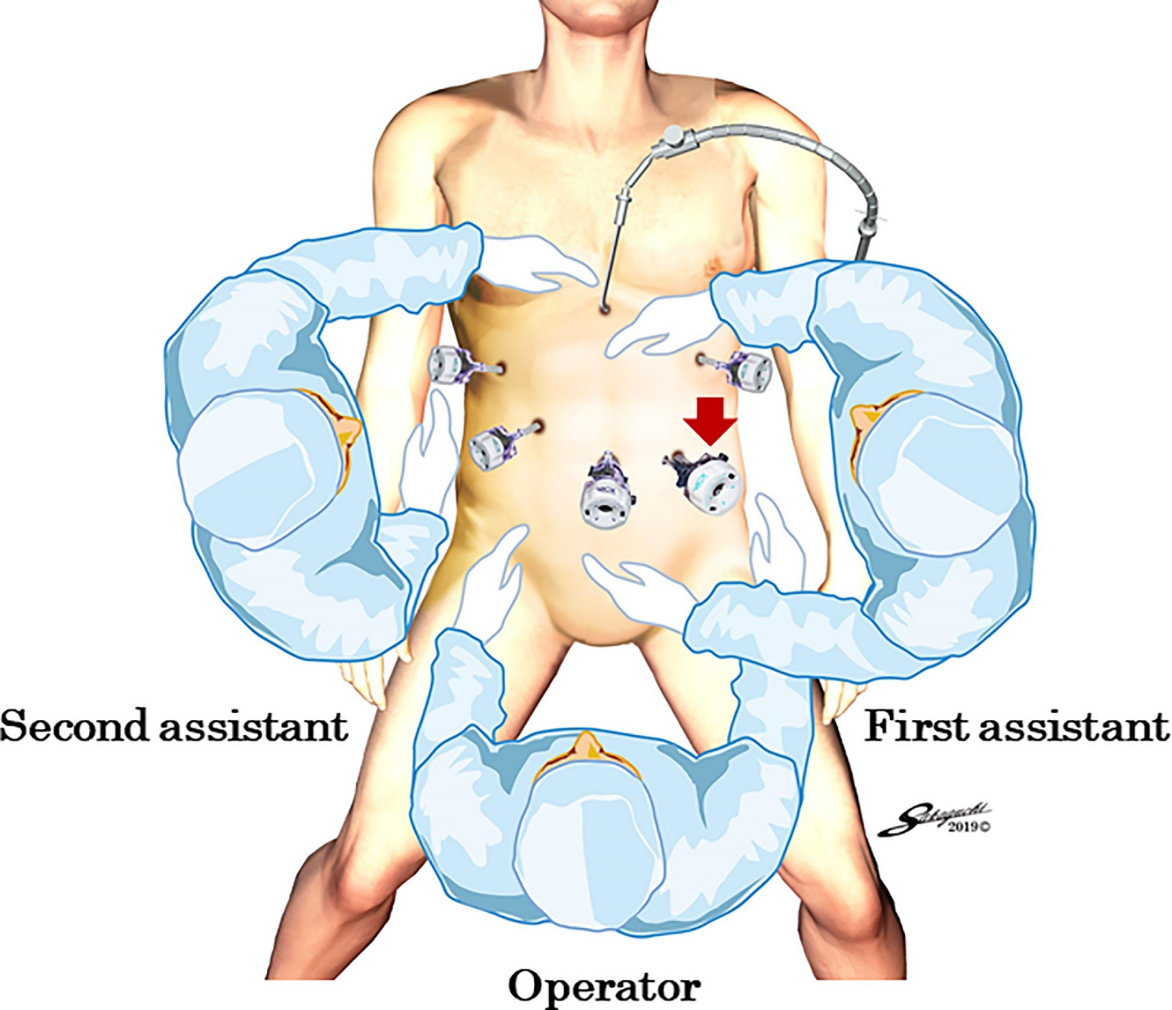
Port placements and positions of the operator and assistants. The stapler is inserted through the left lower port (red arrow), and all staplings are performed by the right hand of the operator positioned between the patient’s legs.

**Fig 2:**
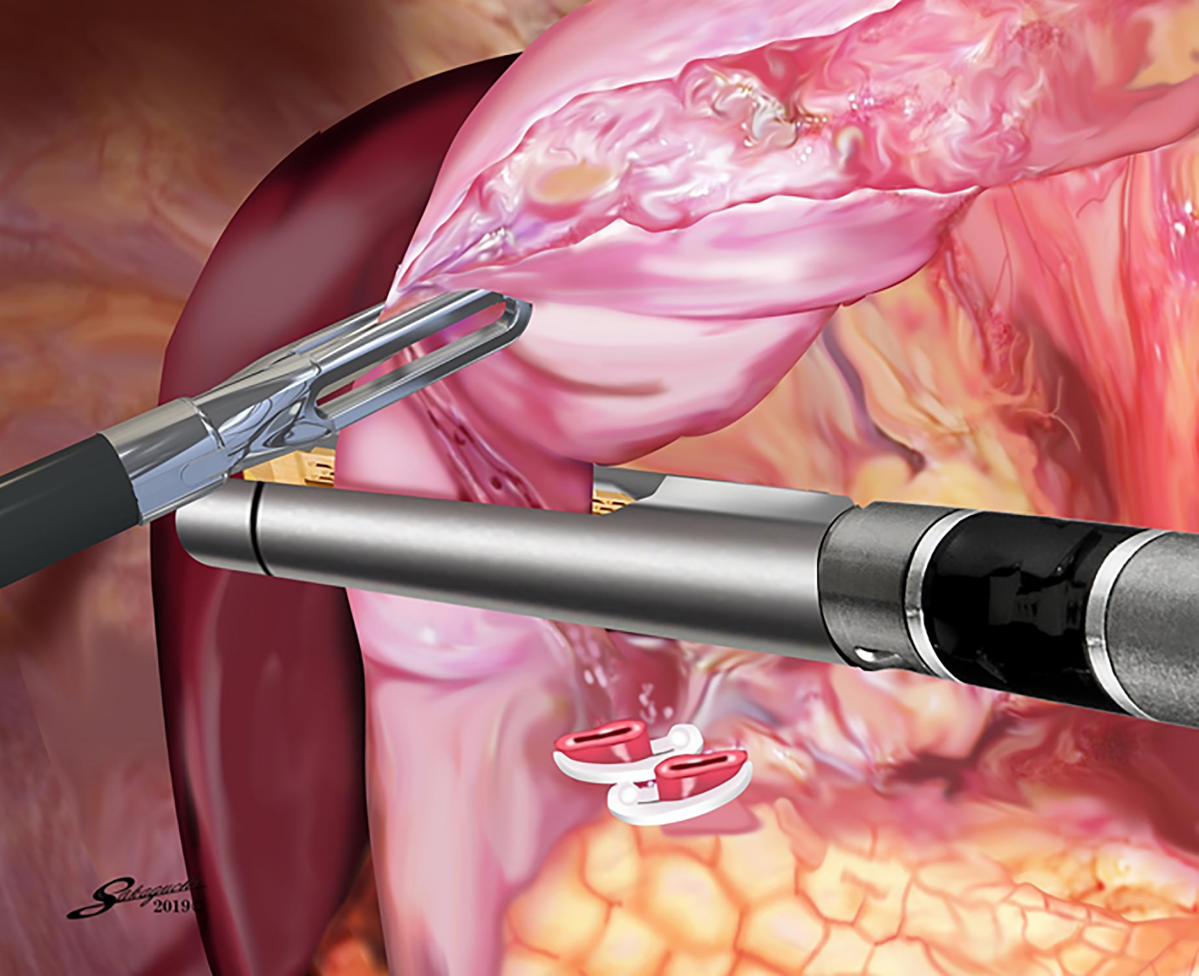
Transection of the duodenal bulb. The operator transects the duodenal bulb using one linear stapler in a posteroanterior direction with the right hand while retracting the greater curvature side of the pyloric ring laterally with the left hand.

Patients were placed in the modified lithotomy position [15]. The operator was positioned between the patient’s legs, with the first assistant manipulating a flexible laparoscope at the patient’s left side and the second assistant at the right side (Fig 1). After five ports were placed in the upper abdomen including the umbilicus, a Nathanson’s retractor was inserted from just below the xiphoid process to elevate the round ligament and the lateral segment of the liver [15]. Because the endoscopic linear stapler was inserted through the left lower port, all staplings were performed by the right hand of the operator positioned between the patient’s legs (Fig 1). LDG with lymphadenectomy based on the Japanese treatment guidelines was performed under a pneumoperitoneum [20]. Dissection of the left greater curvature LNs along the left gastroepiploic artery (No. 4sb), the right greater curvature LNs along the right gastroepiploic artery (No. 4d), and the infrapyloric LNs (No. 6) was performed. The operator transected the duodenal bulb using one linear stapler in a posteroanterior direction with the right hand while retracting the greater curvature side of the pyloric ring laterally with the left hand (Fig 2). The operator then moved to the right side of the patient and dissected the suprapyloric LNs along the right gastric artery (No. 5), the LNs along the root of the left gastric artery (No. 7), the anterosuperior LNs along the common hepatic artery (No. 8a), the celiac artery LNs (No. 9), the proximal splenic artery LNs (No. 11p), and the hepatoduodenal ligament LNs along the proper hepatic artery (No. 12a).The operator then returned to the position between the patient’s legs, dissected the right paracardial LNs (No. 1) and the lesser curvature LNs (Nos. 3a and 3b), and transected the proximal stomach from the greater curvature side to the lesser curvature side with the right hand, referring to preoperative endoscopic markings [21]. The specimen was removed through the extended umbilical wound using a large plastic bag [15]. Pneumoperitoneum was then re-established.

**Fig 3:**
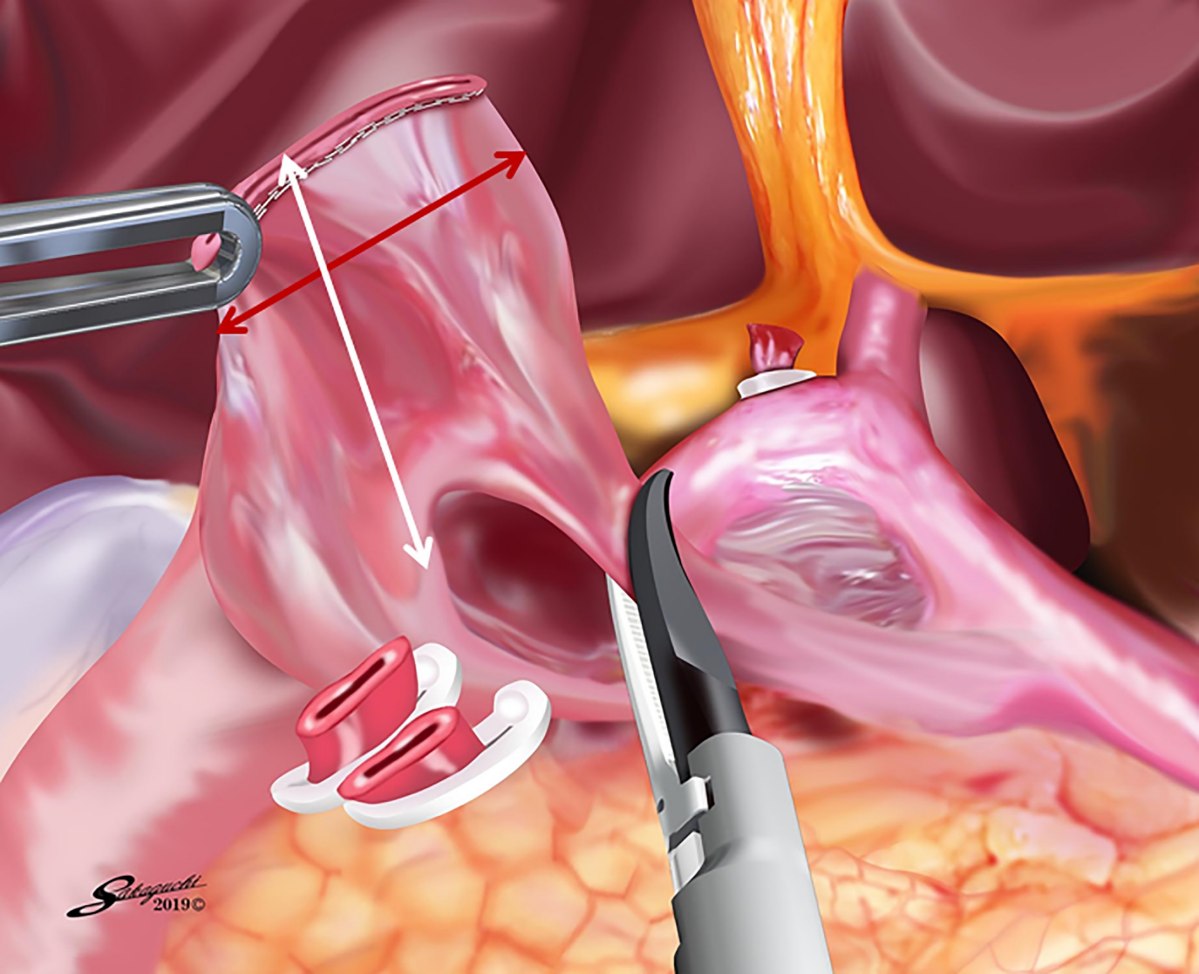
Creation of a space around the posterior wall of the duodenal bulb. A sufficient space for stapler insertion around the posterior wall of the duodenal bulb is created by dissecting the superior duodenal vessels (red line: diameter of the duodenal bulb, white line: length of the duodenal bulb).

**Fig 4:**
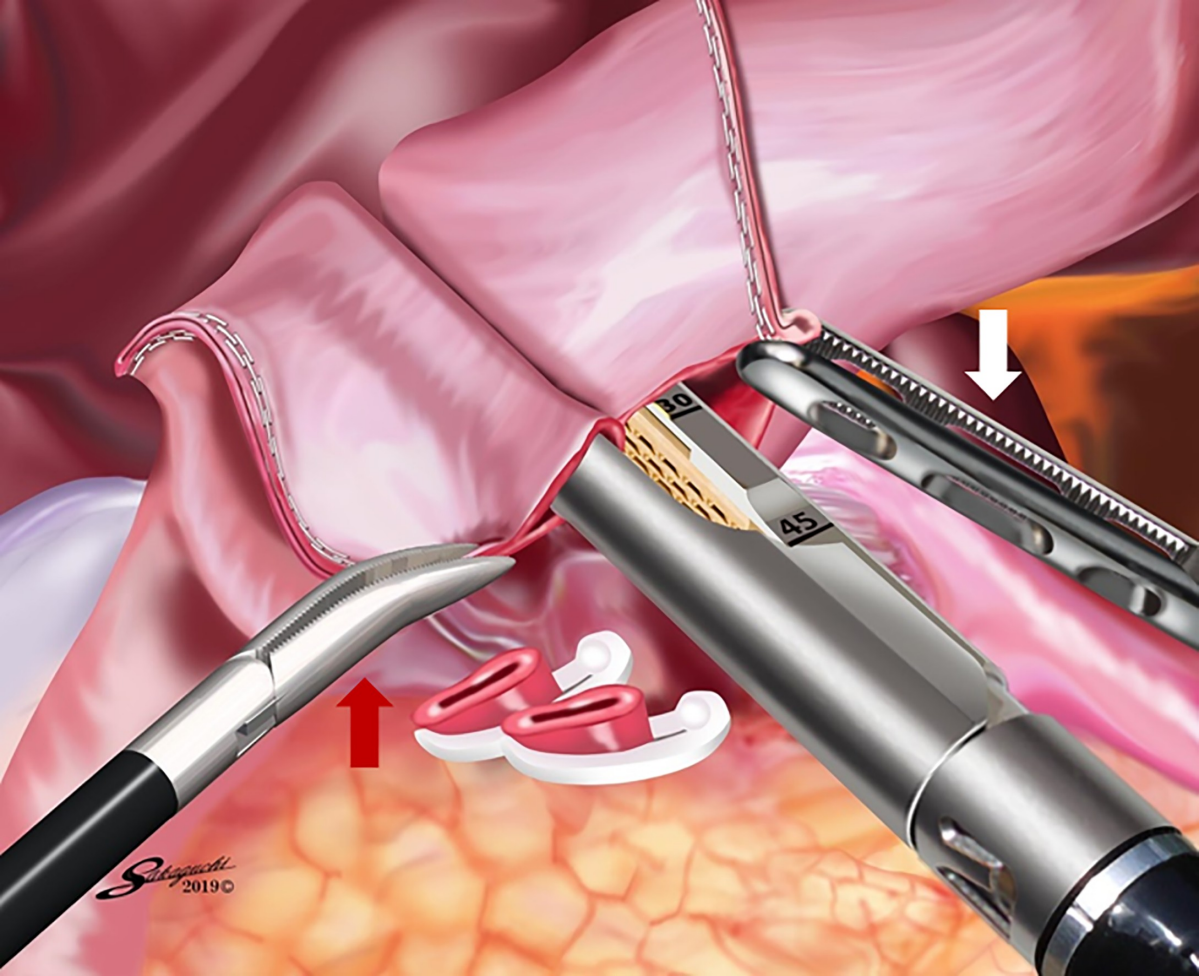
Linear stapling of the posterior walls of the remnant stomach and duodenum. Linear stapling of the posterior walls of the remnant stomach and duodenum without creating a gap using a 45-mm linear stapler with a staple length of 30 mm. The operator performs this stapling with the right hand while retracting the staple line of the duodenum laterally with the left hand (red arrow). The first assistant retracts the staple line of the remnant stomach laterally (white arrow).

**Fig 5:**
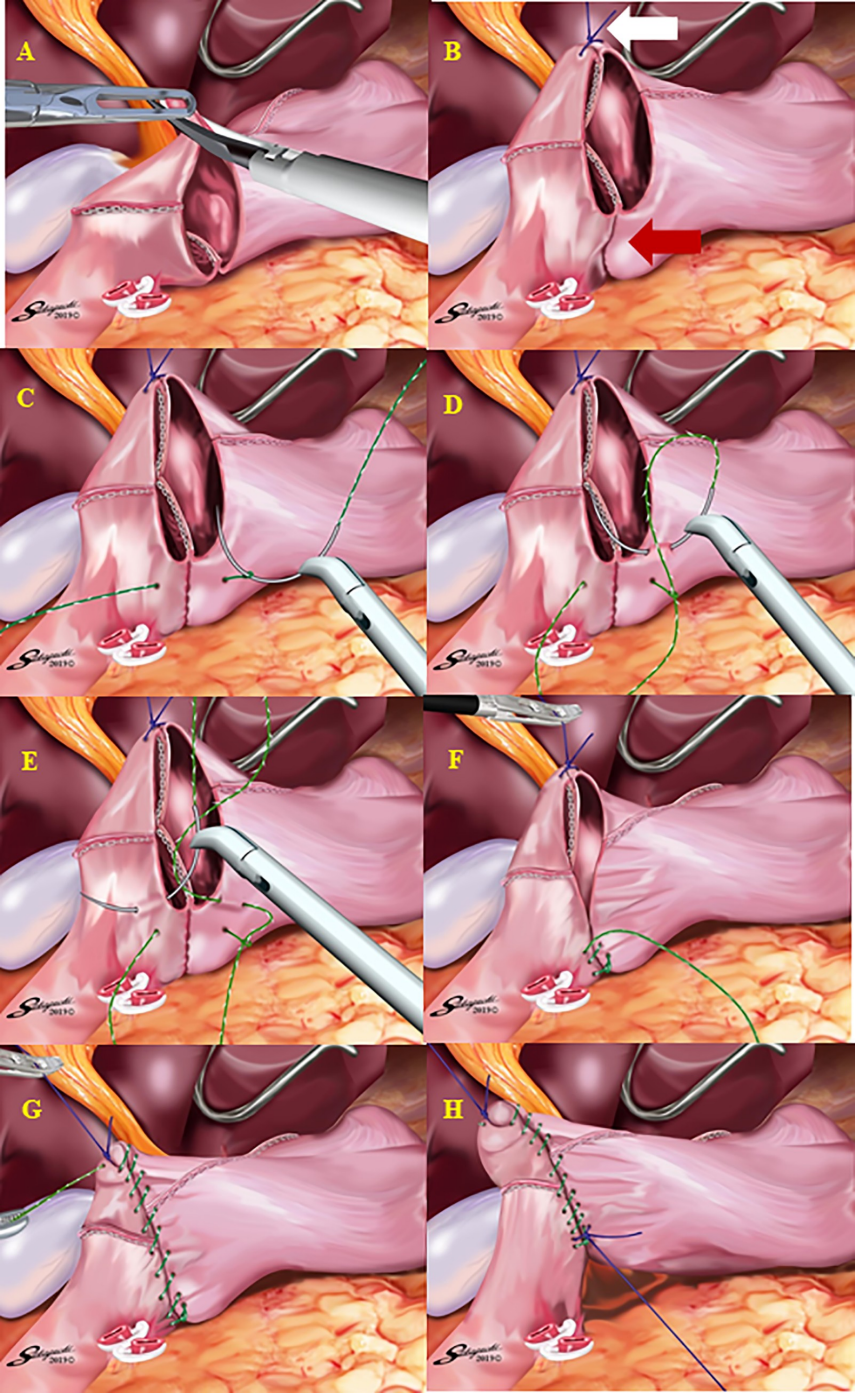
Single-layer, full-thickness hand suturing technique of the stapler entry hole. A The edge of the lesser curvature side of the stapler entry hole is removed. B To create a good view of the greater curvature side (red arrow), the full-thickness knotted suture of 3–0 Prolene at the lesser curvature side (white arrow) is retracted toward the ventral side. C The first full-thickness stitch with a 15-cm-long 3–0 V-Loc 180 is placed on the greater curvature side, and the needle is passed through the loop. D The second and third stitches with the 15-cm-long 3–0 V-Loc 180 are made on the seromuscular layer of the remnant stomach. E The second and third stitches with the 15-cm-long 3–0 V-Loc 180 are made on the full-thickness layer of the duodenum, with the duodenal mucosa being sutured as minutely as possible. F There is no extroversion of the mucosa of the alimentary tract near the greater curvature side. G Single-layer full-thickness continuous suturing of the stapler entry hole is performed with the 15-cm-long 3–0 V-Loc 180 until the suture crosses over the knotted 3–0 Prolene suture on the lesser curvature side. H One or two full-thickness knotted 3–0 Prolene sutures using the extracorporeal slip knot technique (Roeder’s knot) are added at the site near the greater curvature side.

**Fig 6:**
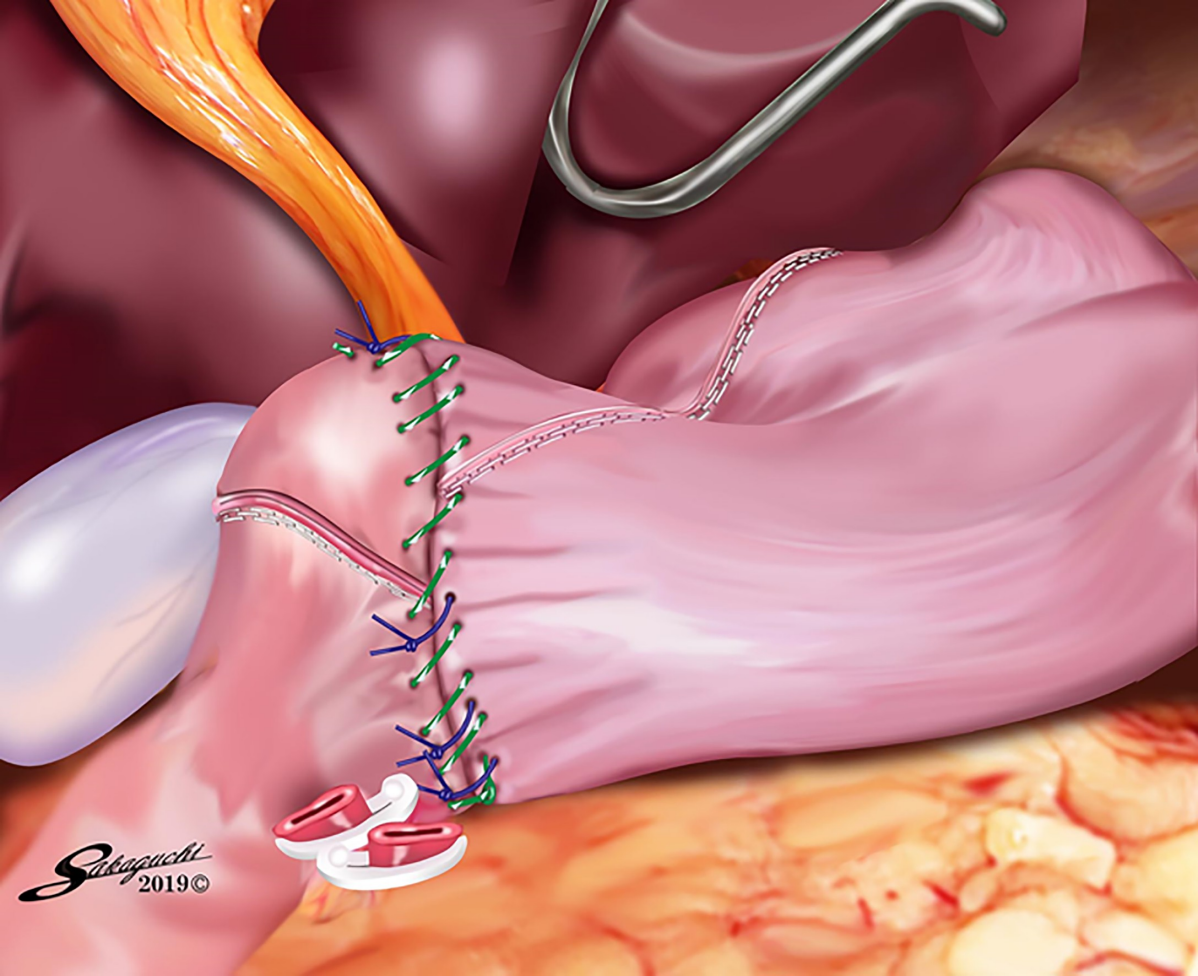
The modified DSG with linear stapling and single-layer hand suturing. The modified DSG with linear stapling and single-layer hand suturing is completed.

After confirming that both the size of the remnant stomach and the length of the duodenal bulb were sufficient, a sufficient space for stapler insertion around the posterior wall of the duodenal bulb was created by dissecting the superior duodenal vessels (Fig 3). Small incisions were made on the greater curvature side of the remnant stomach and the posterior side of the duodenum. The operator inserted a 45-mm linear stapler into the remnant stomach with the right hand, and the first assistant pulled the staple line of the remnant stomach laterally. Then, the operator inserted the stapler into the duodenum with the right hand while pulling the staple line of the duodenum laterally with the left hand. Linear stapling of the posterior walls of the remnant stomach and duodenum without creating a gap was performed, considering the prevention of intraoperative duodenal injury. Fig 4 shows linear stapling with a staple length of 30 mm. The operator closed the stapler entry hole with the right hand using a single-layer full-thickness suturing technique with knotted sutures and a knotless barbed suture after confirming that there was no bleeding in the intraluminal V-shaped staple line, as we previously reported [15]. After removing the edge of the lesser curvature side of the stapler entry hole (Fig 5A), a full-thickness suture of 3–0 Prolene (Ethicon Endo-Surgery, Cincinnati, OH, USA) using the extracorporeal slip knot technique (Roeder’s knot) was placed on the lesser curvature side of the stapler entry hole [15]. After the second assistant retracted this knotted suture toward the ventral side to create a good view of the greater curvature side of the stapler entry hole (Fig 5B), single-layer full-thickness continuous suturing with a 15-cm-long 3–0 V-Loc 180 (VLOCL0604; taper point, 1/2 circle/26 mm; Covidien, Mansfield, MA, USA) was performed from the greater curvature side to the lesser curvature side [15]. The first full-thickness stitch was securely placed on the greater curvature side, and the needle was passed through the loop (Fig 5C) [15]. The second and third stitches were made between the seromuscular layer of the remnant stomach and the full-thickness layer of the duodenum, with the duodenal mucosa being sutured as minutely as possible, to avoid extroversion of the mucosa of the alimentary tract near the greater curvature side (Fig 5D–5F) [15]. This single-layer full-thickness continuous suturing with the 15-cm-long 3–0 V-Loc 180 was performed until the suture crossed over the knotted 3–0 Prolene suture on the lesser curvature side (Fig 5G) [15]. The suture end was cut simply, as short as possible [15]. Routinely, one or two full-thickness knotted sutures of 3–0 Prolene using Roeder’s knot were added at sites near the greater curvature side (Fig 5H) [15]. Similar sutures were made on the site with a broad pitch [15]. Finally, the modified DSG in TLDG was completed (Fig 6).

## Results

**Fig 7:**
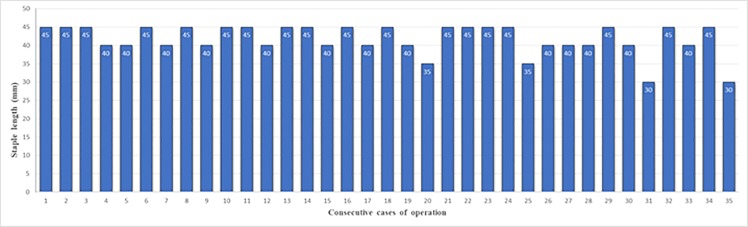
Distribution of staple lengths at linear stapling of the posterior walls of the remnant stomach and duodenum.

**Fig 8:**
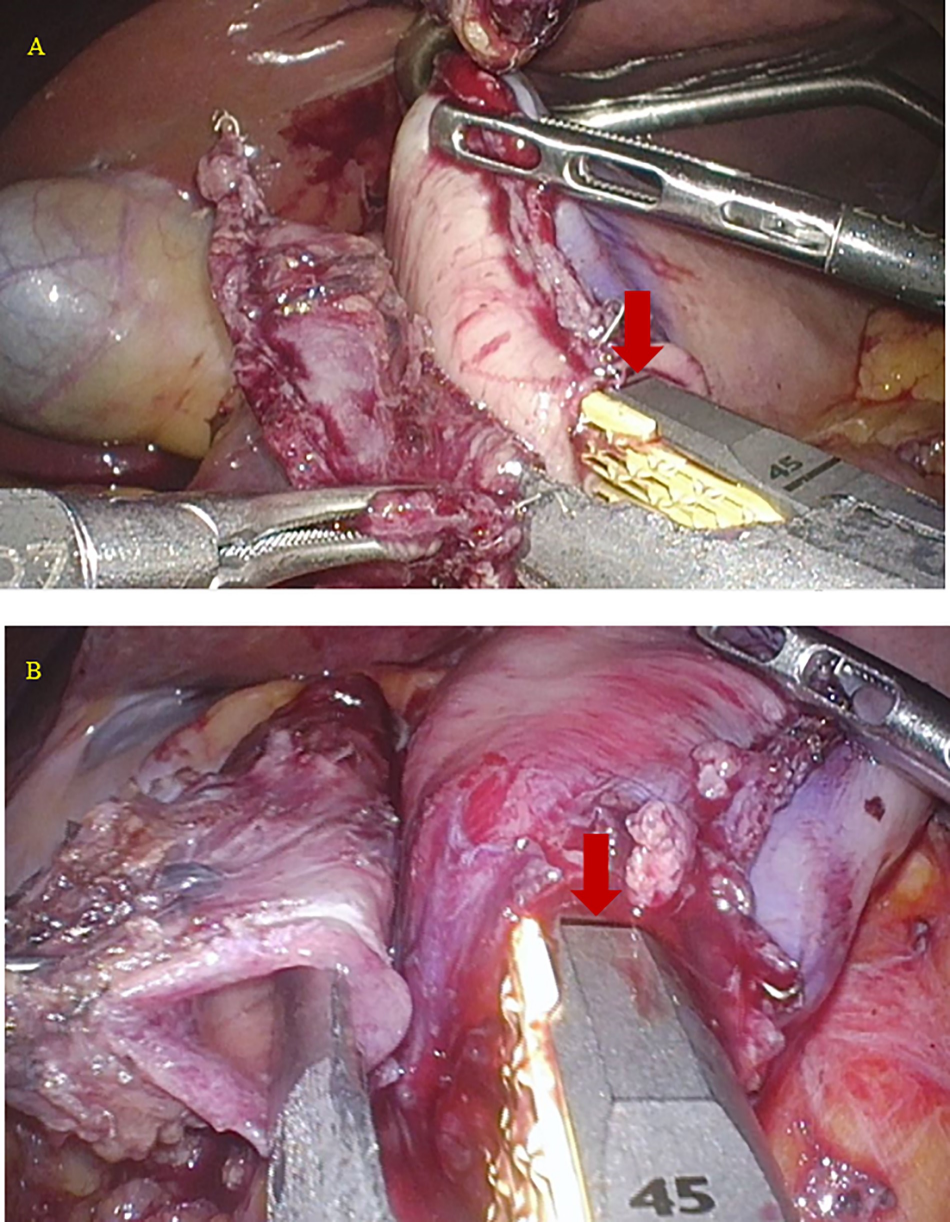
Linear stapling in the patients with a staple length of 30 mm. **A** Linear stapling in Case 31 (red arrow: 30-mm mark on the stapler). **B** Linear stapling in Case 35 (red arrow: 30-mm mark on the stapler).

Patient characteristics and operative findings are described in Table 1. There was no occurrence of intraoperative complications including that of duodenal injury in any of the 35 patients. Distribution of the staple lengths at linear stapling of the posterior walls of the remnant stomach and duodenum is shown in Fig 7. The median staple length was 41.7 ± 4.2 (30–45) mm, and 2 patients had a the staple length of 30 mm (Cases 31 and 35, 5.7%) (Fig 8).

**Table 1:** Patient characteristics and operative findings (n = 35).

Characteristic	Value
Age (years)	66.8 ± 9.0
Sex	
Male	20
Female	15
Body mass index (kg/m^2^)	21.5 ± 2.4
Operative time (min)	252 ± 25
Blood loss (mL)	26.2 ± 5.2
Pathological stage^a^	
IA	25
IB	5
ⅡA	2
ⅡB	1
ⅢB	2
Lymph node dissection^b^	
D1+	30
D2	5
Number of dissected lymph nodes	29 ± 10
Intraoperative complications	0

^a^According to the Japanese classification of gastric carcinoma, 3^rd^ English edition [14].

^b^According to the Japanese gastric cancer treatment guidelines 2014 (ver. 4) [19].

**Fig 9:**
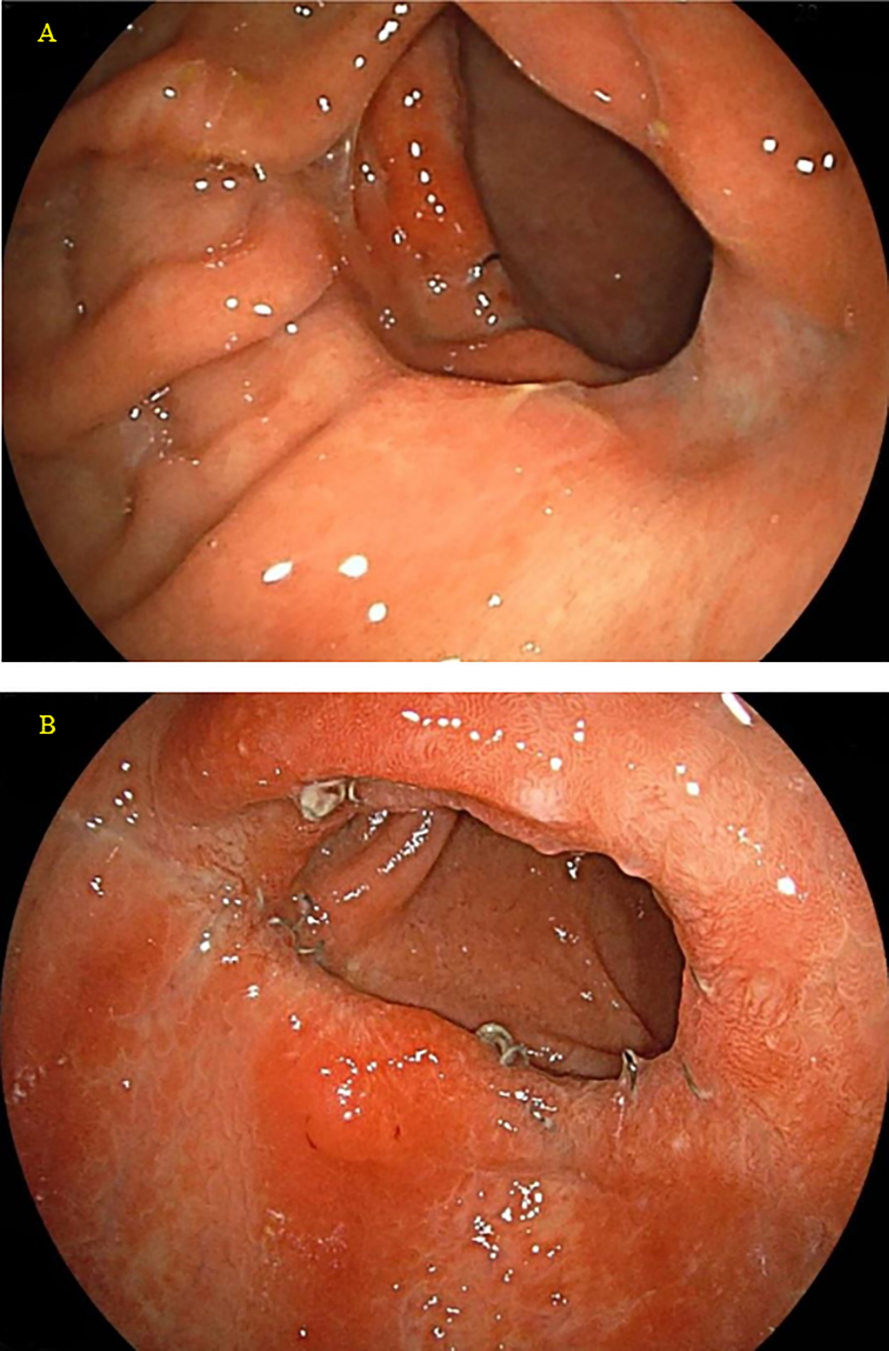
Endoscopic views of the gastroduodenostomy in the patients with a staple length of 30 mm. **A** Endoscopic view 11 months after the operation in Case 31. **B** Endoscopic view 4 months after the operation in Case 35.

The median follow-up duration was 36.5 ± 22.8 (4.3–77.2) months. The overall survival rate in this patient series was 100%. Postoperative complications are shown in Table 2. According to the complications related to the anastomosis, 1 patient (Case 17, 2.9%) had Grade Ⅲa anastomotic hemorrhage at the vertex of the intraluminal V-shaped staple line requiring endoscopic hemostasis on postoperative day 1 [15]. Otherwise, no other postoperative anastomosis-related complications including anastomotic stenosis or leakage occurred in any of the 35 patients. In addition, no patients were diagnosed as having dumping syndrome. All patients underwent endoscopic examination in the outpatient care after discharge. Endoscopic views of the gastroduodenostomy in the patients with a staple length of 30 mm are shown in Fig 9 (Cases 31 and 35).

**Table 2:** Postoperative complications (n = 35).

Complication	Number (%)
Anastomotic leakage	
Grade Ⅱ^a^	0
Grade Ⅲa/b	0/0
Anastomotic stenosis	
Grade Ⅱ	0
Grade Ⅲa/b	0/0
Anastomotic hemorrhage	
Grade Ⅱ	0
Grade Ⅲa/b	1 (2.9)/0
Pancreatic fistula	
Grade Ⅱ	1 (2.9)
Grade Ⅲa/b	0/0
Intraabdominal abscess	
Grade Ⅱ	0
Grade Ⅲa/b	0/0
Intestinal obstruction	
GradeⅡ	0
Grade Ⅲa/b	0/0

^a^Grading of complications was based on the Clavien-Dindo classification [18]

## Discussion

In conventional open distal gastrectomy, B-Ⅰ reconstruction is considered difficult to perform when the remnant stomach is small or the length of the duodenal bulb is short. Therefore, to perform B-Ⅰ reconstruction in TLDG safely and securely, not only a sufficient size of the remnant stomach but also a sufficient length of the duodenal bulb is necessary. Several techniques of intracorporeal gastroduodenostomy, including the DSG, have been described. Takiguchi et al. reported B-Ⅰ reconstruction using the Albert-Lembert method with laparoscopic hand-sewn techniques [22]. Although this method does not require a long duodenal bulb length, it does require adequate suturing skill. In the DSG, after transecting the duodenum in a posteroanterior direction and creating a sufficient space for stapler insertion around the posterior wall of the duodenal bulb, the posterior walls of the remnant stomach and duodenum are anastomosed. Thereafter, the stapler entry hole is closed using the linear stapler. Thus, the DSG necessitates a longer duodenal bulb length than that of B-Ⅰ reconstruction using the Albert-Lembert method with laparoscopic hand-sewn techniques.

Okabe et al. reported the presence of intraoperative duodenal injury in the DSG due to stapler insertion during linear stapling of the posterior walls of the remnant stomach and duodenum [11]. It was considered that this complication might occur when the diameter of the duodenal bulb was relatively short and that one cause might be that stapling was performed with the left hand of the assistant positioned at the patient’s left side under the guidance of the operator positioned at the patient’s right side. In the present patient series, the operator was positioned between the patient’s legs while stapling. At result, the operator could perform linear stapling of the posterior walls of the remnant stomach and duodenum with the right hand while retracting the duodenum laterally with the left hand.

Kim et al. [2] and Lee et al. [12] reported the occurrence of postoperative anastomotic stenosis in the DSG. Similar to intraoperative duodenal injury, it was considered that this complication might occur when the diameter of the duodenal bulb was relatively short because the staple length at linear stapling of the posterior walls of the remnant stomach and duodenum was not sufficient and the stapler entry hole was closed using the linear stapler. Leyba et al. described gastrojejunostomy using the linear stapler in laparoscopic gastric bypass for morbid obesity in which after a 45-mm linear stapler was introduced 18 mm into the stomach and jejunum and fired, the stapler entry hole was closed using the double-layer continuous suturing technique. They reported the incidence of stenosis to be 2.5% in their series [23]. In contrast, Bendewald et al. reported gastrojejunostomy using the linear stapler in laparoscopic gastric bypass for morbid obesity in which after a 30-mm linear stapler was introduced 20 mm into the stomach and jejunum and fired, the stapler entry hole was closed using the double-layer continuous suturing technique, and the incidence of stenosis in this series was 6.0% [24]. In the present patient series, linear stapling of the posterior walls of the remnant stomach and duodenum without creating a gap using a 45-mm linear stapler was performed, considering the prevention of intraoperative injury. There was no intraoperative duodenal injury, and the median staple length was 41.7 ± 4.2 (30–45) mm. Thus, a staple length of at least 30 mm could be attained, and the stapler entry hole was closed using a single-layer full-thickness hand suturing technique in this patient series. As a result, there was no incidence of postoperative anastomotic stenosis.

Noshiro et al. [8] reported the presence of anastomotic leakage and intraabdominal abscess around the anastomosis. Among their initial 71 patients undergoing DSG, 6 experienced anastomotic leakage, 2 developed an intraabdominal abscess around the anastomosis, and in all of these patients, the affected site was the greater curvature side of the stapler entry hole [8]. They offered two possible reasons for these complications. First, closing the greater curvature side of the entry hole using the linear stapler was uncertain because this side tended to roll back behind the stapler [8]. Second, the extroverted gastroduodenostomy sometimes directly contacted the pancreatic head after dissection of the infrapyloric LNs (No. 6), so even minimal leakage of pancreatic juice might be activated after contact with the mucosa of the alimentary tract [8]. In the present patient series, after a good view of the greater curvature side of the stapler entry hole was created, single-layer full-thickness continuous hand suturing of the entry hole with a knotless barbed suture was initiated from the greater curvature side, avoiding extroversion of the mucosa of the alimentary tract near this side [15]. Routinely, one or two full-thickness knotted sutures were added at sites near this side [15].

At result, there was no incidence of either anastomotic leakage or intraabdominal abscess around the anastomosis in this patient series.

Dumping syndrome is considered to be one of the concerns in B-Ⅰ reconstruction following distal gastrectomy. Kanaya et al. [5] reported that the delta-shaped gastroduodenostomy could prevent dumping syndrome because of the occurrence of brief stasis due to duodenal twisting at the site of the delta-shaped anastomosis. There was no incidence of dumping syndrome in the present patient series.

This study had some limitations. First, it was a single-institution retrospective study with a small sample size. Second, the median follow-up period was relatively short. Third, this reconstruction technique was not compared to other anastomotic methods. Fourth, because most patients in this series had a body mass index < 25 kg/m^2^, the utility of this technique was not evaluated in very obese patients. A prospective randomized study in a larger sample size of patients with a variety of body mass indexes needs to be performed.

In conclusion, we suggest that a modified DSG consisting of linear stapling and single-layer hand suturing performed by an operator positioned between the patient’s legs can be one option for B-Ⅰ reconstruction following TLDG because it can aid in the prevention of both intraoperative duodenal injury and postoperative anastomotic stenosis.

## Supporting information

S1 Video Single-layer, full-thickness hand suturing technique of the stapler entry hole. (MP4)
